# PIN-PMN-PT Single Crystal 1-3 Composite-based 20 MHz Ultrasound Phased Array

**DOI:** 10.3390/mi11050524

**Published:** 2020-05-21

**Authors:** Wei Zhou, Tao Zhang, Jun Ou-Yang, Xiaofei Yang, Dawei Wu, Benpeng Zhu

**Affiliations:** 1 School of Optical and Electronic Information, Huazhong University of Science and Technology, Wuhan 430074, China; davidzhou@hubcomm.com (W.Z.); D201880657@hust.edu.cn (T.Z.); oyj@hust.edu.cn (J.O.-Y.); yangxiaofei@hust.edu.cn (X.Y.); 2 Jingzhou Vocational College of Technology, Jingzhou 434000, China; 3 State Key Laboratory of Transducer Technology, Chinese Academy of Sciences, Shanghai 200050, China; 4 State Key Laboratory of Mechanics and Control of Mechanical Structures, Nanjing University of Aeronautics and Astronautics, Nanjing 210016, China

**Keywords:** PIN-PMN-PT, 1-3 composite, high frequency, phased array

## Abstract

Based on a modified dice-and-fill technique, a PIN-PMN-PT single crystal 1-3 composite with the kerf of 12 μm and pitch of 50 μm was prepared. The as-made piezoelectric composite material behaved with high piezoelectric constant (d_33_ = 1500 pC/N), high electromechanical coefficient (*k_t_* = 0.81), and low acoustic impedance (16.2 Mrayls). Using lithography and flexible circuit method, a 48-element phased array was successfully fabricated from such a piezoelectric composite. The array element was measured to have a central frequency of 20 MHz and a fractional bandwidth of approximately 77% at −6 dB. Of particular significance was that this PIN-PMN-PT single crystal 1-3 composite-based phased array exhibits a superior insertion loss compared with PMN-PT single crystal and PZT-5H-based 20 MHz phased arrays. The focusing and steering capabilities of the obtained phased array were demonstrated theoretically and experimentally. These promising results indicate that the PIN-PMN-PT single crystal 1-3 composite-based high frequency phased array is a good candidate for ultrasound imaging applications.

## 1. Introduction

Over the past few decades, due to its safety, convenience, and efficiency, ultrasound has attracted significant attention in biomedical research and clinical diagnosis [1,2,3,4,5,6]. To meet the requirement of high resolution imaging and precise manipulation, the operational frequency of biomedical ultrasound needs to be greater than 20 MHz. For such applications, high frequency ultrasound transducer, which plays an important role in producing and receiving ultrasound signal, is indispensable [7,8,9,10,11,12,13]. Currently, to the best of our knowledge, single element transducers and linear arrays are widely used [14,15,16,17]. Only a few studies have been carried on the high-frequency ultrasound phased array [18,19,20,21], though it is very useful in biomedical imaging by providing electronic-beam-focusing and steering capabilities. Furthermore, there are even fewer researches on composite-material-based high-frequency ultrasound phased arrays, despite the numerous benefits of composite piezoelectric materials (lower acoustic impedance, higher electromechanical coefficient, broader bandwidth, etc.). As is known, the pitch of a phased array is much less than that of a linear array at the same operating frequency, and the kerf of a composite material must be small enough to avoid spurious modes. The above facts give rise to smaller size in both kerf and each element, which poses even greater challenges for piezoelectric material composite preparation and high frequency (≥20 MHz) phased array fabrication.

In recent years, single-crystal piezoelectrics have been developing a revolutionary solution to substitute traditional PZT ceramics for ultrasonic transducer applications [22,23,24]. Recently, PMN-PT single-crystals have been used to manufacture high frequency (≥20 MHz) phased arrays and some attractive results have been found [19,20]. However, the drawback of PMN-PT single-crystals is their relatively low-temperature usage range [22]. To make the devices maintain a better temperature stability, a high Curie temperature (Tc) ternary piezoelectric single crystal [Pb(In_1/2_Nb_1/2_)O_3_-Pb(Mg_1/3_Nb_2/3_)O_3_-PbTiO_3_, abbreviated as PIN-PMN-PT], which maintains a similar electromechanical and piezoelectric performance (d_33_~1500 pm V^–1^, k_33_ > 90%) to a PMN-PT single crystal, has been developed [25,26]. In previous studies, the PIN-PMN-PT single crystal and its composite have been proven to be promising candidates for high frequency single element transducer fabrication [7,26,27]. Additionally, compare with piezoelectric single crystal itself and its 2-2 composite, the single crystal 1-3 composite usually exhibits higher coupling coefficient and suitable acoustic impedance. Hence, it is of great interest to demonstrate the feasibility of the development of a PIN-PMN-PT single crystal 1-3 composite-based high frequency (≥20 MHz) ultrasound phased array.

In this work, a modified dice-and-fill technique for PIN-PMN-PT single crystal 1-3 composite preparation is introduced. The design, fabrication, and characterization of a 20 MHz side-looking 48-element high frequency ultrasound phased array are presented. Furthermore, to demonstrate the imaging capability of this obtained device, wire phantom imaging experiments are carried out based on a commercial Verasonics Vantage 128 System.

## 2. Design and Fabrication

**Figure 1:**
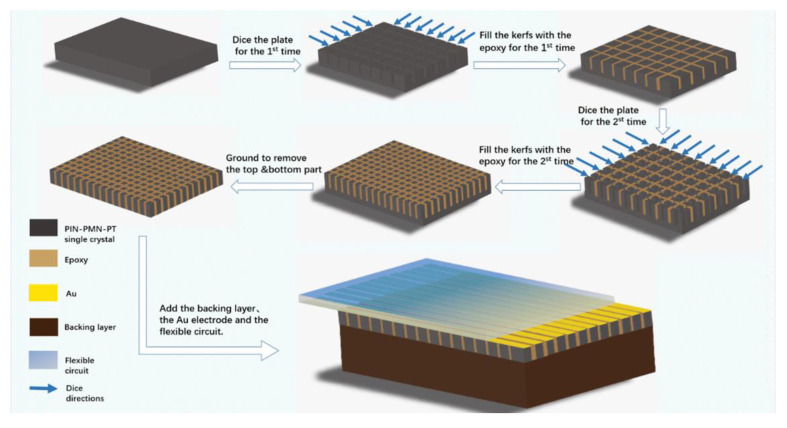
Schematic of the modified dice-and-fill method used for phased array fabrication.

In our fabrication, the [1]-oriented single crystal with a composition of 0.27PIN-0.45PMN-0.28PT was selected. Generally, for the fabrication of phased array with central frequency around 20 MHz, the pitch should be less than λ (wavelength in water, 75 μm). In fact, taking into consideration the technology difficulty, the pitch in our design is 50 μm. Figure 1 describes the 1-3 composite fabrication process, during which a modified dice-and-fill method was adopted to prevent the collapse and cracking of the elements. To obtain a high precision kerf width when dicing the PIN-PMN-PT single crystal, a 10-μm-thick nickel/diamond blade with a DAD323 dicing saw (DISCO, Saitama, Japan) was chosen. First, the PIN-PMN-PT wafer was diced from two perpendicular directions with a 100 μm pitch and 80 μm depth to form periodic rods. The kerf was around 12 μm because of the dicing saw’s vibration during dicing process. Then, the kerfs were filled with low viscosity epoxy (Epo-Tek-301, Epoxy Technology, Billerica, MA, USA), and the trapped bubbles were removed in vacuum for 15 min. After epoxy solidification at 40 °C for 10 h, the PIN-PMN-PT rods in the epoxy matrix were further diced in the two previous perpendicular directions with the same dicing pitch and depth to form equal areas. The newly formed kerfs were epoxy filled and cured again. Finally, to grind off the excess single-crystal and epoxy, a PIN-PMN-PT single crystal 1-3 composite with a 50 μm pitch and 80 μm depth was obtained. The main piezoelectric properties of the PIN-PMN-PT single-crystal 1-3 composite are listed in Table 1. With the Archimedes principle, we can know that the density of this kind of composite is 4510 kg/m^3^. In addition, the longitude velocity was measured to be 3600 m/s. Consequently, using the equation of Z = ρc, the acoustic impedance can be calculated to be 16.2 MRayl. In addition, the piezoelectric coefficient d_33_ (1500 pC/N) was tested by a d_33_ meter (ZJ-6A, Institute of Acoustics, Chinese Academy of Sciences, Beijing, China), and the loss (0.023) was evaluated by a LCR meter (Agilent, Englewood, CO, USA, 4263B). The electromechanical coupling factor (0.81) was calculated using resonance and anti-resonance frequencies, which were measured by an Agilent 4294A electric impedance analyzer (Agilent Technologies, Englewood, CO, USA). These excellent properties imply that this PIN-PMN-PT single crystal 1-3 composite is competent for ultrasound device application.

**Figure 2:**
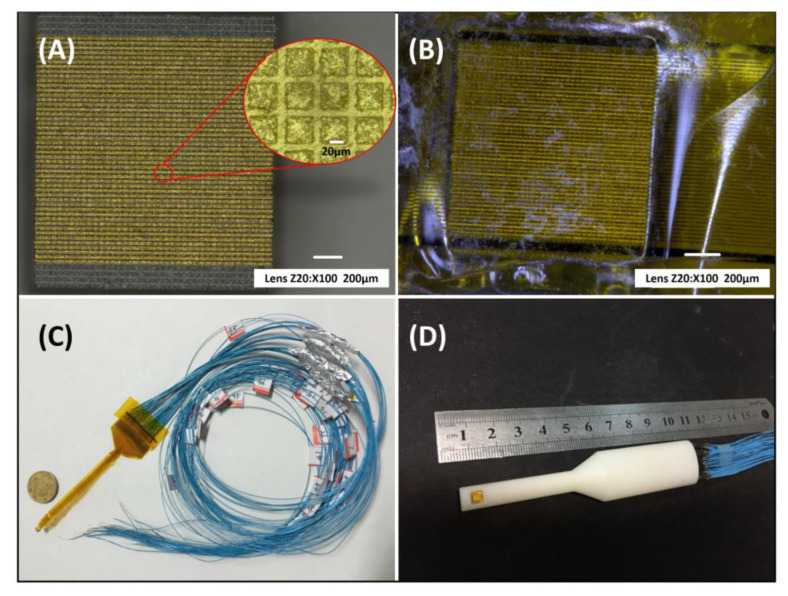
Photographs of (**A**) Au patterned PIN-PMN-PT single crystal 1-3 composite (**B**) with Flexible Circuit and (**C**) electrical connections; (**D**) the side-looking phase array in 3D-printed housing.

After sputtering Au/Cr (150 nm/100 nm) layers on both sides of the 1-3 composite, E-solder 3022 was added as the backing layer, and lithography was used to pattern 48 elements with a 38 μm width, 3 mm length, and 12 μm kerf, as presented in Figure 2A. Then, a piece of 30 μm polyimide-based flexible circuit(Kapton, DuPont, Wilmington, DE, USA) with gold patterns was attached to the front of the array using Epo-Tek 301 epoxy for electrical connections, as shown in Figure 2B,C. Actually, as long as the thickness of Epo-Tek 301 epoxy is in a suitable range, it can have a good adhesion; otherwise, it has no effect on conductivity. Because the flexible circuit (Kapton) has a specific acoustic impedance of 3.4 MRayl, it can also act as a matching layer for the phased array. Lastly, the 48-element side-looking phased array was encapsulated in a 3D-printed housing, as depicted in Figure 2D.

**Figure 3:**
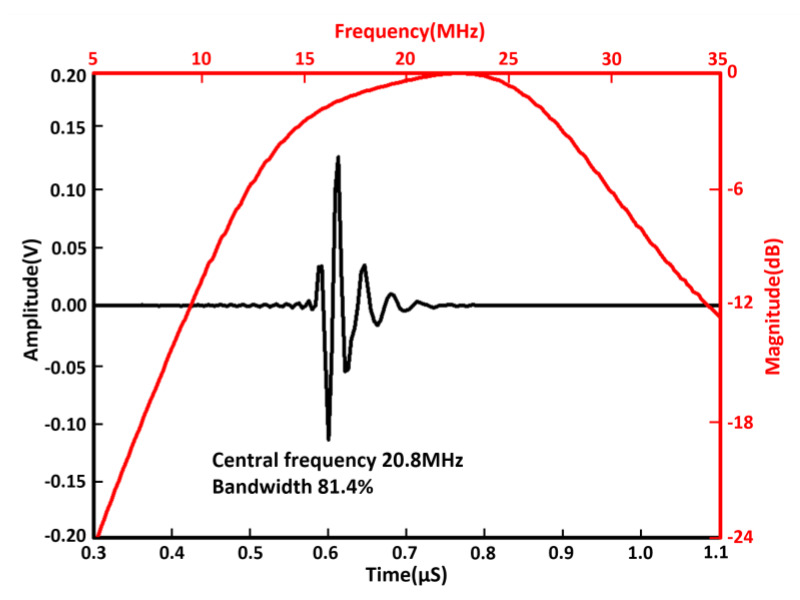
The simulated pulse-echo waveform and frequency spectrum of the transducer using the PiezoCAD software.

To simulate the acoustic performance of our phased array, the Krimholz, Leedom, and Mattaei (KLM) equivalent circuit-based software package PiezoCAD (Sonic Concepts, Woodinville, WA, USA) was employed. The acoustic design parameters for piezoelectric single crystal 1-3 composite phased array transducer are listed in Table 2. For our design, the thickness of polyimide-based flexible circuit, PIN-PMN-PT single crystal 1-3 composite, and E-solder 3022 are 30 μm, 80 μm, and 2.5 mm, respectively. Figure 3 shows the simulated pulse-echo waveform and frequency spectrum of this phased array. It can be easily seen that the operational frequency is 20.8 MHz and bandwidth at −6 dB is 81.4%.

## 3. Characterization and Discussions

**Figure 4:**
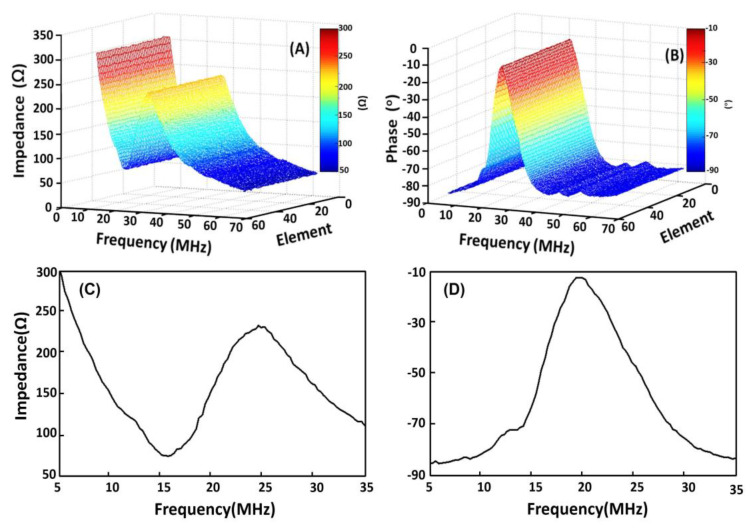
(**A**) Electrical impedance magnitude and (**B**) phase as a function of frequency for 48 elements. (**C**) Electrical impedance magnitude and (**D**) phase as a function of frequency for the 24th element.

Figure 4 shows the measurements of the electrical impedance and phase for the array elements using the impedance analyzer Agilent 4294A. It is clear that no critical differences can be found among the 48 elements, indicating that all the elements are uniform. The peaks in the phase curves are located at 20.4 MHz, suggesting that the operational frequency for the array is approximately 20 MHz. According to the IEEE standard [28], the thickness mode of the electromechanical coupling coefficient (kt) for each array element can be determined from the following equation:

(1) $$k_{t}^{2}=\frac{\pi }{2}\frac{f_{r}}{f_{a}}\tan\left(\frac{\pi }{2}\frac{f_{a}-f_{r}}{f_{a}}\right),$$

where $f_{r}$ and $f_{a}$ are the resonant and antiresonant frequencies, respectively. Substituting the appropriate values into Equation (1), $k_{t}$ was calculated to be 0.81. This value is similar to those reported in the literature [26,29].

**Figure 5:**
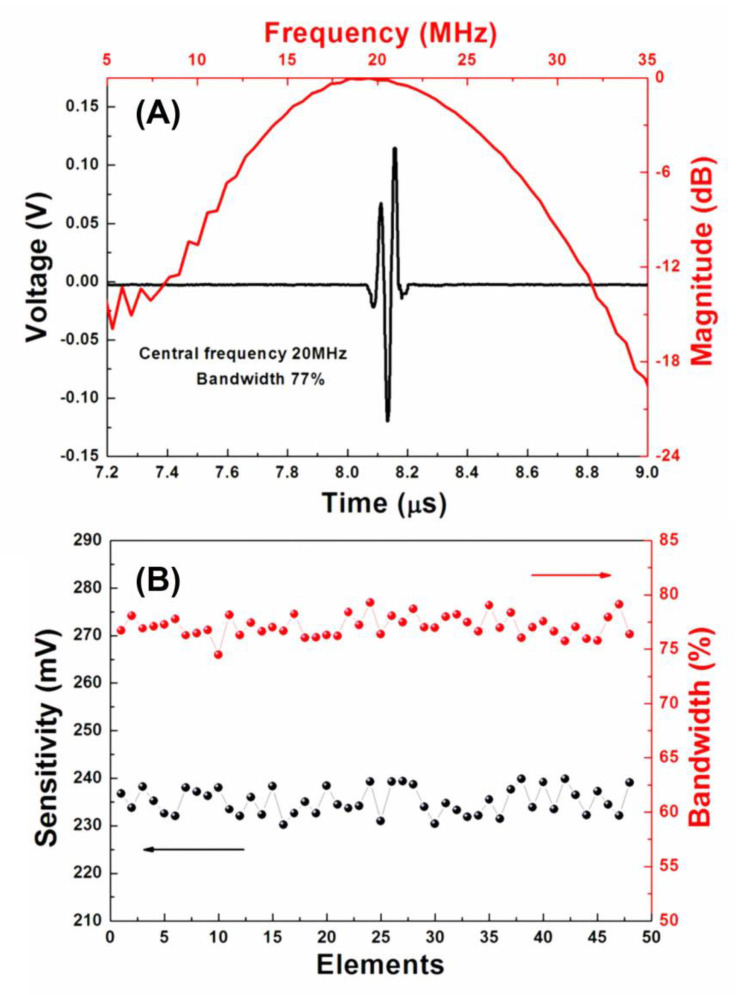
(**A**) Measured pulse-echo response performance of random element; (**B**) sensitivity and bandwidth of the pulse-echo signal for each element.

The pulse-echo response of the representative element of the phased array transducer was acquired in a deionized water tank at room-temperature using a pulser receiver (5900PR, Panametrics, Inc., Waltham, MA, USA). The echo was reflected by an X-cut quartz plate target, the waveform of which was recorded using a 1-GHz oscilloscope (LC534, LeCroy Corp., Chestnut Ridge, NY, USA). As shown in Figure 5A, the random array element exhibits a peak-to-peak echo amplitude of 0.235 V. The measured central frequency and bandwidth at −6 dB are 20 MHz and 77%, respectively, which is closed to the simulated result. It should be noted that, as described in Figure 5B, the array exhibits a good uniformity in acoustic performance (sensitivity: 0.235V ±2.1%, bandwidth: 77% ± 3%).

The insertion loss (IL) and crosstalk were measured using several cycles of a sinusoid pulse produced by a Sony/Tektronix AFG2020 arbitrary function generator. For the insertion loss (IL) measurement, the amplitude of an echo signal reflected by the quartz target was received by the excited phased-array element. The exciting waveform is a tone burst of 20-cycle sinusoidal wave with the amplitude of the driving signal and the center frequency. An oscilloscope connected with the excited phased-array element was set to 1 MΩ and 50 Ω to test the receiving and transmitting signal, respectively. In consideration of the compensation for the attenuation in water (2.2 × 10^−4^ dB mm^–1^ × MHz^2^) and the loss caused by the imperfect reflection from the quartz target (1.9 dB) [1], the IL was calculated using

(2) $$\mathrm{IL}=20\log\frac{V_{R}}{V_{T}}+1.9+2.2\times 10^{-4}\times 2d\times f_{c}^{2},$$

where $f_{c}$ is the center frequency, *V_T_* and *V_R_* are the transmitting and receiving amplitudes, and d is the distance between the target and transducer. The IL value of the phased array elements was measured to be −19.7 dB, which is superior to those of PMN-PT single crystal- and PZT-5H-based high frequency (≥20 MHz) phased arrays [18,19,20]. This phenomenon is probably related to the high Curie Temperature of PIN-PMN-PT single crystal. The heat induced by lapping and dicing in fabrication process produces nearly no effluence to its electrical properties. Therefore, PIN-PMN-PT single crystal 1-3 composite array element can sustain a high piezoelectric performance. For the crosstalk measurement, the amplitude of an echo signal reflected by the quartz target was received by the nearest neighbor element of the excited one. According to the method reported in Reference [18], the measured crosstalk between the nearest neighbor array elements at the center frequency was −38 dB.

**Figure 6:**
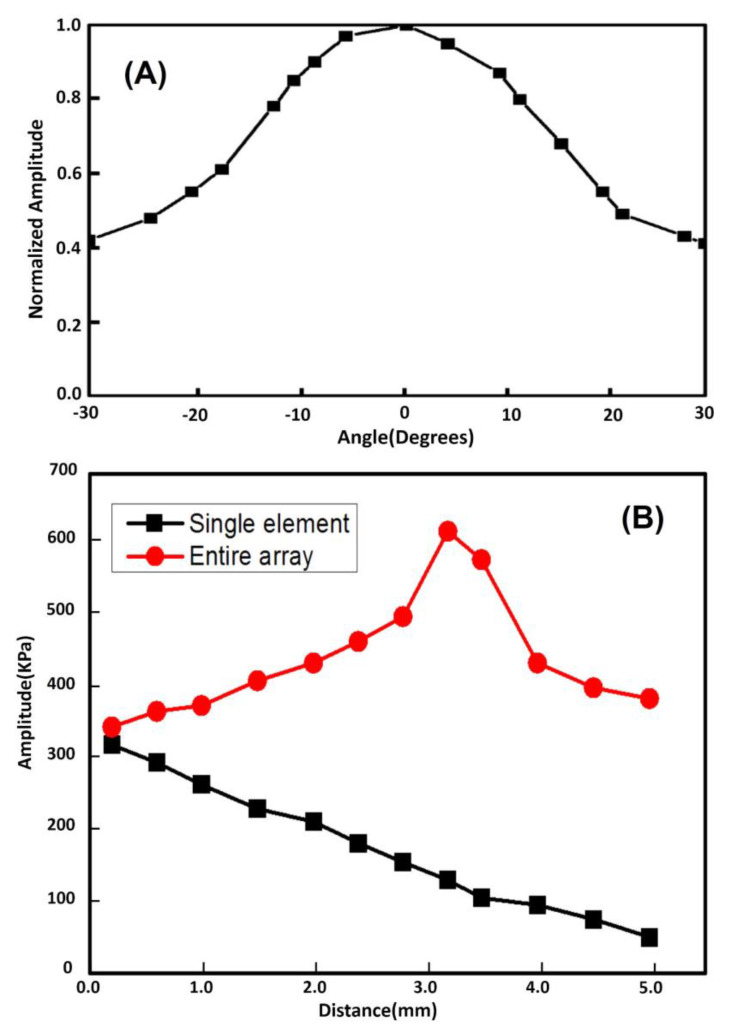
(**A**) Measured one-way azimuthal directivity responses of a representative array element (24th element). (**B**) The acoustic output in the axial direction of a representative array element (24th element) and the entire array.

In order to measure the one-way azimuthal directivity response, a representative array element (24th element) was excited. The amplitude of the time-domain response of the element at discrete angular positions was acquired by a piezoelectric hydrophone with 0.2 mm diameter (Precision Acoustic, Dorset, UK). As can be seen in Figure 6A, for our phased array, the measured −6 dB directivity is approximately ±21°. Meanwhile, the similar result was reported in Reference [20], a −6 dB directivity of approximately ±20° for a 20 MHz phased array. For the evaluation of the acoustic output characteristics of the transducer, both the acoustic pressure of a representative element (24th element) and the entire array in the axis direction were measured by this commercial needle piezoelectric hydrophone. As shown in Figure 6B, the maximum acoustic pressure for an element near the surface is about 323 KPa, and the maximum acoustic pressure of the entire array near the focusing position is 625 KPa. These results indicate that this PIN-PMN-PT single crystal 1-3 composite-based phase array has the potential for ultrasound imaging application.

**Figure 7:**
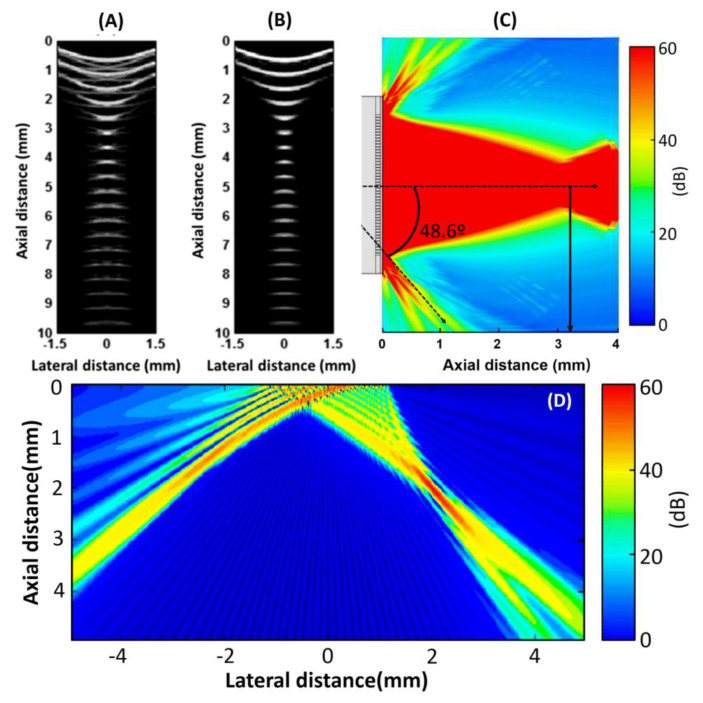
Point spread function phantom-imaged (**A**) without and (**B**) with apodization. (**C**) Simulated phased array acoustic pressure of emission mode when the steering angle is 0°. (**D**) Simulated acoustic beam generated by the 48-element phased array when the steering angle is 45°and the focal distance is 3 mm.

To predict the imaging performance of the obtained phased array, the Field II Ultrasound Simulation Program was utilized. In the simulation, an aperture consisting of 48 active elements of the array was assumed to test the focusing capability of the phased array. Multiple point targets located evenly between 0–10 mm along the aperture central axis were first created. The simulated result is presented in Figure 7A in a 60 dB dynamic range and the focal distance is about 3.2 mm. The imaging performance can be improved by applying apodization, as shown in Figure 7B. Because the pitch of the fabricated array is 50 µm and the half-wavelength of the 20 MHz sound wave in water is 37.5 µm, the side-lobes thus appear at arcsin(37.5/50), which is 48.6°, and the result is consistent with the simulation result, as shown in Figure 7C. When the beam is steered at the angle of 45°, the grating lobe shares quite a large part of the energy, as Figure 7D. The image results also indicate that the ultrasound energy is concentrated at about 3 mm along the axis. The image resolutions of the points deteriorate when they are off-axis or move away from the 3-mm distance.

**Figure 8:**
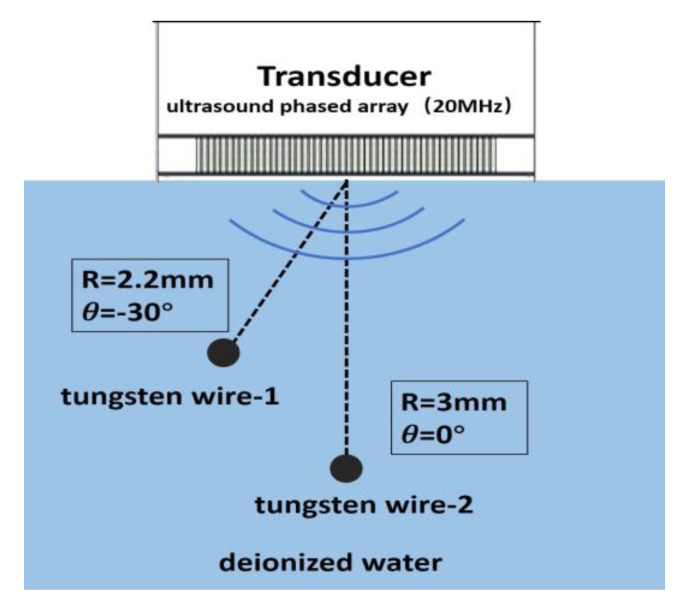
Schematic diagram of tungsten wire phantom in deionized water.

**Figure 9:**
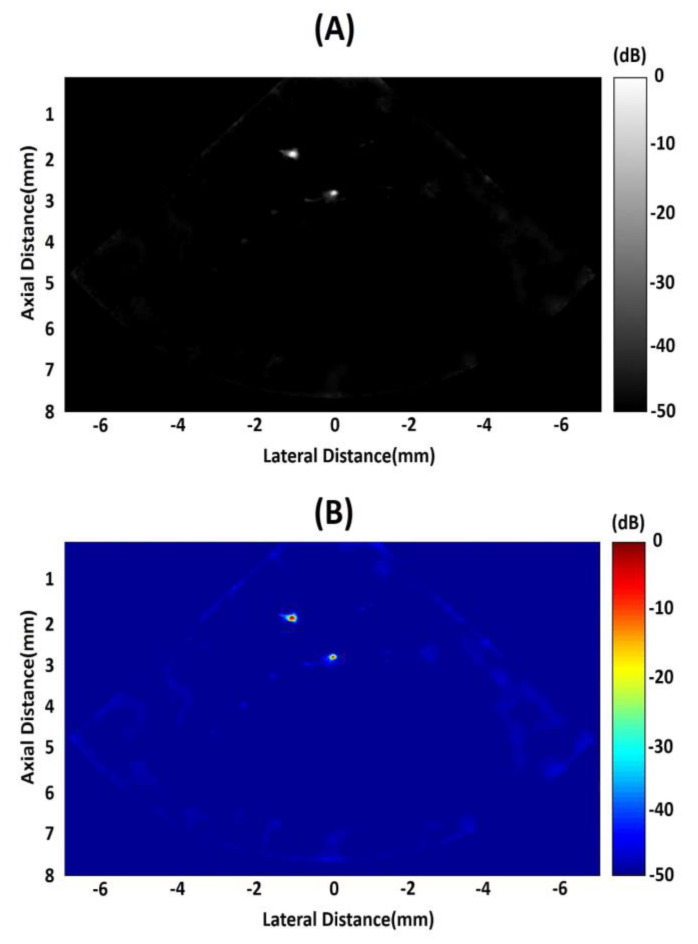
(**A**) Acquired image of custom-made fine-wire phantom. (**B**) Pseudo-color image of custom-made fine-wire phantom.

**Figure 10:**
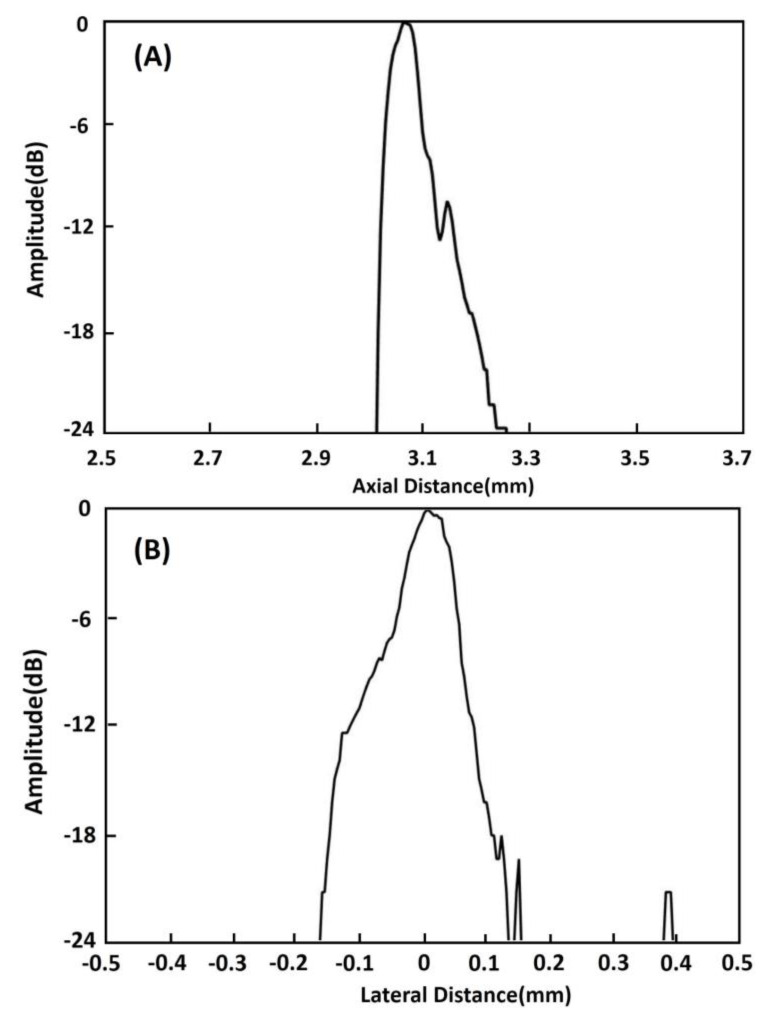
(**A**) Axial and (**B**) lateral line spread functions for the second wire of the wire phantom.

A commercial Verasonics Vantage 128 System (Verasonics, Inc., Kirkland, WA, USA) was utilized to determine the imaging capability of this 20 MHz phased array. In our imaging experiment, two kinds of phantoms were employed. One is 20 μm-diameter tungsten wire in water and the other is 200 μm-diameter copper wire in PDMS (polydimethylsiloxane). In order to determine the axial and lateral spatial resolution of the array transducer, two 20-μm-diameter tungsten wires were positioned at different places, as shown in Figure 8, and immersed in a tank with deionized water. In Figure 9A,B, with gray scale and color scale in 50 dB dynamic range, it can be observed that the two wires are clearly visible. For the second wire, which is located at the focal point, it obviously has a better resolution. We can obtain the theoretical resolutions using the following equations [30]:

(3) $$R_{A}=PL/2,$$

(4) $$R_{L}=F\#\times \lambda ,$$

where $R_{A}$ and $R_{L}$ are the axial and lateral spatial resolutions, respectively. PL is the −6 dB spatial pulse length of the received echo(measured PL of 150 μm), F# is the F-number of the array transducer (1.25), and λ is the sound wavelength in the transmitting medium (75 μm in the water). Therefore, the theoretical axial resolution is 75 μm and theoretical lateral resolution is 94 μm. Plots of the axial and lateral line spread functions for the second wire are shown in Figure 10A,B. The measured spatial resolutions at −6 dB are approximately 77 μm and 125 μm in the axial and lateral directions, respectively. These values are in accordance with those theoretical values.

**Figure 11:**
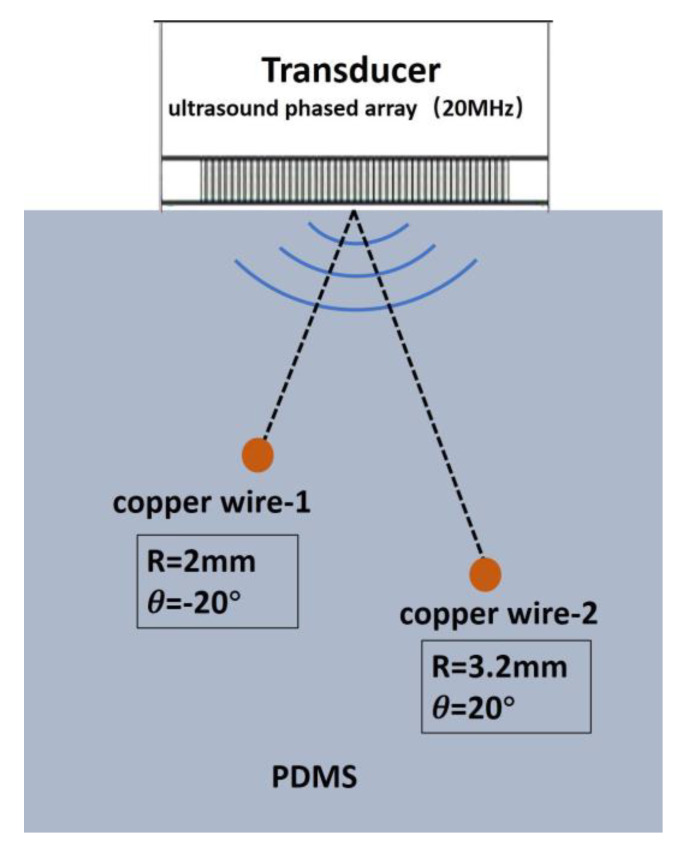
Schematic diagram of copper wire phantom in PDMS.

**Figure 12:**
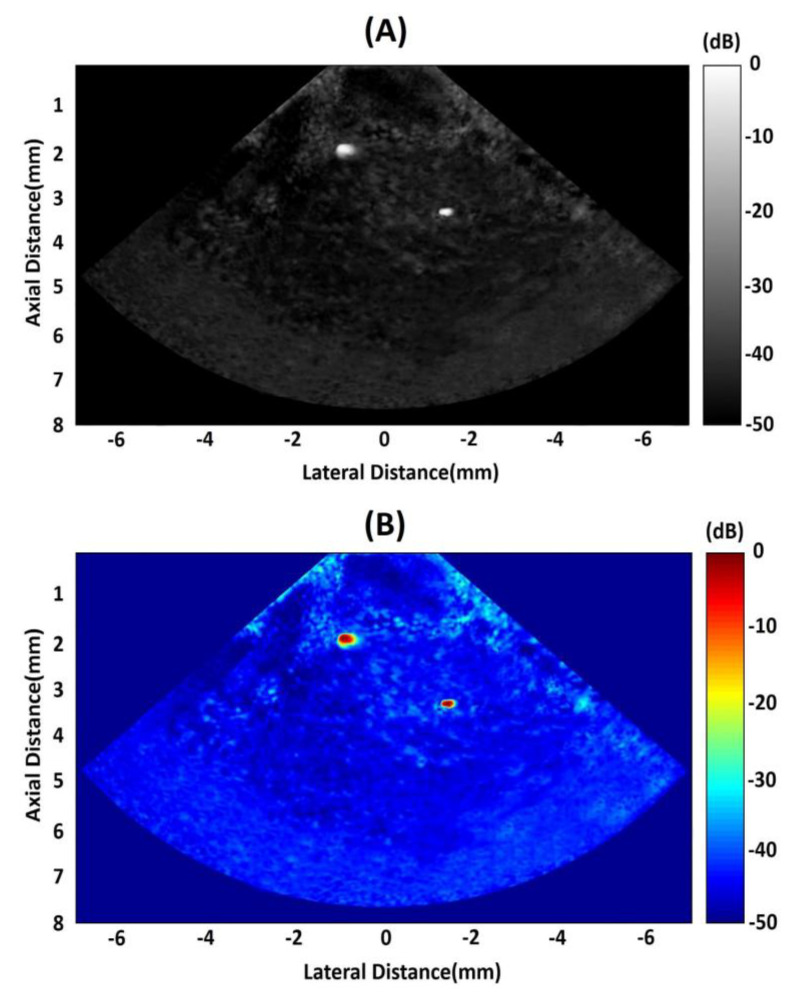
(**A**) Copper wires phantom image. (**B**) Pseudo-color image of copper wires phantom.

As shown in Figure 11, two 200 μm diameter copper wires are placed in the PDMS at different positions. The purpose is to demonstrate the capability of this PIN-PMN-PT single crystal 1-3 composite-based phase array to detect solid structures embedded in tissue. In Figure 12A,B, with gray scale and color scale in 50 dB dynamic range, it is clear to find that the two wires can be easily observed. Compared with the first wire, the second one’s resolution is superior, which demonstrates that this obtained high frequency phased array has an excellent beam steering performance.

## 4. Conclusions

Based on a modified dice-and-fill technique, a PIN-PMN-PT single crystal 1-3 composite with high piezoelectric constant (d_33_ = 1500 pC/N), high electromechanical coefficient (k_t_ = 0.81), and low acoustic impedance (16.2 Mrayls) was prepared. Utilizing this kind of composite, a 20 MHz 48-element side-looking high frequency phased array with central frequency of 20 MHz and −6 dB bandwidth of 77% was successfully fabricated, which was confirmed by the electric impedance resonance curve and the pulse-echo response. Of particular significance was that this PIN-PMN-PT single crystal 1-3 composite-based phased array exhibits a superior insertion loss compared with PMN-PT single crystal and PZT-5H-based 20 MHz phased arrays. The focusing and steering capabilities of the obtained phased array were demonstrated theoretically and experimentally. Furthermore, when using such a phased array, wire phantom images in water and PDMS can be achieved. These promising results suggest that the PIN-PMN-PT single crystal 1-3 composite-based high frequency phased array is competent for biomedical ultrasound imaging in the future.

## Figures and Tables

**Table 1:** Properties of piezoelectric single crystal 1-3 composite.

Piezoelectric material	0.27PIN-0.45PMN-0.28PT
Polymer	Epoxy 301
Longitude velocity	3600 ms^−1^
Density	4510 kg/m^3^
Acoustic impedance	16.2 MRayls
Piezoelectric constant	1500 pC/N
Electromechanical coupling coefficient	0.81
Loss tangent	0.023

**Table 2:** The acoustic design parameters for piezoelectric single crystal 1-3 composite phased array transducer.

Layer	Material	Acoustic Impendence/MRayl	Thickness/mm
Matching layer	Polyimide-based flexible circuit	3.4	0.03
Piezoelectric layer	Single crystal 1-3 composite	16.2	0.08
Backing layer	E-solder 3.22	5.92	2.5
